# A Complex Interplay between Wnt/****β****-Catenin Signalling and the Cell Cycle in the Adult Liver

**DOI:** 10.1155/2012/816125

**Published:** 2012-09-03

**Authors:** Angélique Gougelet, Sabine Colnot

**Affiliations:** ^1^INSERM, U1016, Institut Cochin, 75014 Paris, France; ^2^CNRS, UMR8104, 75014 Paris, France; ^3^Université Paris Descartes, Sorbonne Paris Cité, 75270 Paris, France

## Abstract

Canonical Wnt signalling, governed by its effector **β**-catenin, is known for a long time as playing an important role in development, tissue homeostasis, and cancer. In the liver, it was unravelled as both an oncogenic pathway involved in a subset of liver cancers and a physiological signalling identified as the “zonation-keeper” of the quiescent liver lobule. This duality has encouraged to explore the role of canonical Wnt in liver regeneration and liver-cell proliferation mainly using murine genetic models of **β**-catenin overactivation or inactivation. These studies definitely integrate Wnt signalling within the hepatic network driving regeneration and proliferation. We will review here the current knowledge concerning the mitogenic effect of Wnt, to switch on its specific role in the liver, which is quiescent but with a great capacity to regenerate. The duality of **β**-catenin signalling, associated both with liver quiescence and liver-cell proliferation, will be brought forward.

## 1. Introduction

Since 1982 and the initial discovery of Int1 (Wnt1a) being an oncogene in murine breast cancers, Wnt signalling has been strongly associated with cancer and therefore with cell proliferation [1]. Firstly described as triggering G1 phase progression through Cyclin D1 and c-Myc transcriptional inductions [2–4], it now clearly appears that the interplay between the cell cycle and Wnt signalling is more complex, specific for cell and tissue contexts and not only transcriptional ([5], for review).

**Figure 1:**
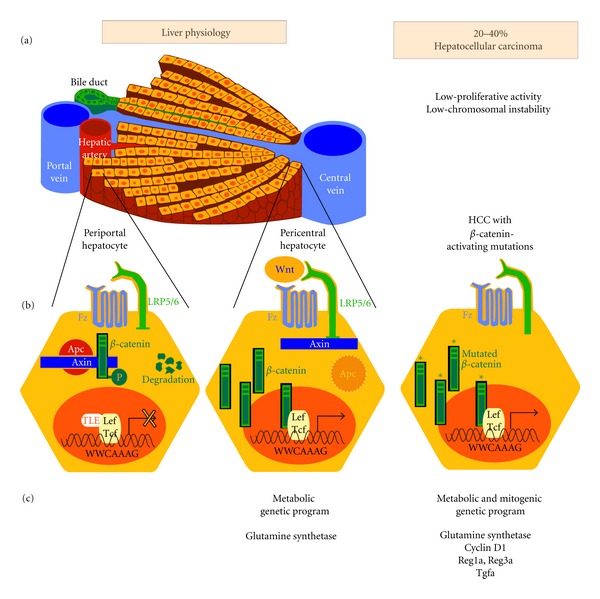
Wnt signalling in the adult quiescent liver and in CTNNB1-mutated HCCs. (a) The liver-cell plate and its portocentral organization; (b) the periportal hepatocyte is deprived of Wnt signalling, due to its high amount of Apc, allowing the destruction complex to be efficient to degrade *β*-catenin. The pericentral hepatocyte has a Wnt-dependent *β*-catenin signalling, while in HCCs it is constitutively activated due to mutations in phosphorylation residues in CTNNB1; (c) the output of the transcriptional *β*-catenin is metabolic in the pericentral hepatocyte, while it is both metabolic and mitogenic in HCCs.

The Wnt signalling consists either in a canonical or a noncanonical pathway and only the better characterized canonical pathway will be depicted here ([6], for review). *β*-catenin is the main effector of the canonical signalling (Figure 1). In cells not submitted to Wnt ligands, the cytosolic *β*-catenin is continuously ubiquitinylated for degradation through sequential phosphorylations by the caseine kinase 1 (CK1) and the glycogen synthase kinase 3 (GSK3). This occurs within a so-called “destruction complex” scaffolded by the tumor suppressors AXINS. The tumor suppressor APC (Adenomatous polyposis coli) is required within this destruction complex for an efficient degradation of *β*-catenin. Upon Wnt ligand binding to its frizzled receptor and LRP5/6 coreceptor at the membrane, a cascade of events impedes GSK3 kinase activity, through LRP5/6-dependent sequestration of GSK3 within endosomal vesicles [7]. The ensuing accumulation of *β*-catenin triggers its nuclear translocation and its association with a Lef/Tcf DNA-binding partner. This leads to the transcription of a genetic program specific for the temporal, spatial, and tissue contexts ([8], for review). Mutations in critical partners of the pathway, that is, *β*-catenin gene- (CTNNB1-) activating mutations, loss-of-function mutations in APC, AXIN1, or AXIN2 genes, induce a constitutive activation of *β*-catenin signalling and are found in a large number of human cancers ([9], for review).

## 2. Wnt and the Cell Cycle: An Overview

How Wnt/*β*-catenin signalling is mitogenic has been widely explored in many experimental systems and has been shown to occur at distinct levels ([5], for review). 

### 2.1. Wnt Transcriptional and Nontranscriptional Effects during the G1 Phase

Entering S phase and DNA replication is a key decision that forces cells to divide, and it has to be regulated during G1 phase. Indeed, most signalling pathways that regulate cell proliferation exert their effects in G1 ([10], for review). Cyclin D is an important regulator of the checkpoints allowing G1-to-S progression, including the inactivation through phosphorylation of the Retinoblastoma (Rb) complex, increasing Cyclin E levels. At the opposite, growth inhibitory signals inhibit cyclin D (and cyclin E) through p21 and p27 accumulation, thereby leading to entry into quiescence [10].

 As expected, Cyclin D1 has been one of the first transcriptional target genes of *β*-catenin described in colorectal cancer cell lines [3, 4]. But most important was the involvement of c-Myc as a Wnt transcriptional target [2], because this transcription factor has a dual role in G1 phase by promoting Cyclin D [11] and repressing p21 and p27 [12]. This importance has been emphasized by the fact that in the intestine, c-Myc ablation fully rescues Apc loss-driven tumorigenesis [13]. Interestingly, it is not the case in the liver [14], consistent with the fact that c-Myc is surprisingly not a transcriptional target of *β*-catenin in that tissue [15–17].

But Wnt signalling-mediated GSK3 inhibition not only induces transcriptional changes. GSK3 phosphorylates and destabilizes other substrates than *β*-catenin, among which are direct regulators of G1 progression, such as cyclin D1, cyclin E1, and c-myc [18–20]. Lastly, GSK3 is also a key inhibitor of cell growth occurring in G1, during which cells increase their protein levels otherwise they would become smaller after cell division. Similarly to the classical IGF/AKT pathway, Wnt/Gsk3 signalling activates the TOR pathway to stimulate protein translation, including that of Cyclin D1: indeed GSK3 activates TSC2, an inhibitor of the TOR pathway [21].

### 2.2. Microtubule Dynamics during Mitosis

During mitosis, cells divide both chromosomes and cell components into daughter cells. Thereby it is a phase in which predominate subcellular mechanics, with transcription and translation being dampened [5]. Nevertherless, some aspects of the mitotic program that is, the microtubule (MT) dynamics, spindle formation, and centrosome division can be partly ascribed to Wnt components, even if it is not clearly assessed that these features would be controlled by Wnt/*β*-catenin signalling (reviewed in [5]).

Briefly, MT dynamics are regulated by Wnt signalling, even without any mitosis [22]. The tumor suppressor APC has been the first component of Wnt signalling to be associated to the mitotic spindle, and this is required for proper chromosome segregation: therefore, Apc loss was shown to lead to chromosomal instability [23, 24], and can also induce polyploidy ([25], this is of interest for the physiologically polyploid liver, reviewed by Gentric et al., in this issue). AXIN2 also associates to the mitotic spindle [26]. Lastly, AXIN2, *β*-catenin, and GSK3 accumulate at the centrosomes, which align the mitotic spindle. Therein, they regulate MT growth ([5], for review).

### 2.3. Cell Cycle Impact on Wnt Signalling Amplitude

Wnt signalling influences the cell cycle but conversely the cell cycle has an impact on Wnt signalling. It had been observed that *β*-catenin levels oscillate with the cell cycle, and peak in mitosis [27]. Similarly, the expression of some *β*-catenin targets (Lgr5, AXIN2, but not c-myc) peaks at G2/M [28]. This phenomenon has recently found a molecular explanation through LRP6 phosphorylation, required for LRP6 to respond to Wnt ligands. This phosphorylation is primed by the cyclin-dependent kinase 14 (cdk14), which associates with and is regulated by the G2/M cyclin Y. The optimal response of LRP6 coreceptor to Wnts is therefore cell cycle dependent, and consequently the maximal activation of *β*-catenin occurs at G2/M [29].

## 3. Wnt Signalling in the Liver

### 3.1. Wnt in Liver Oncogenesis

In 1998, the link between Wnt signalling and the liver was initially established, through the demonstration that *β*-catenin activating mutations occur in 20 to 40% of hepatocellular carcinoma (HCC) [30, 31]. HCC is an heterogenous disease that differs both by its risk factors (Viral hepatitis B and C, alcohol abuse, metabolic liver disease, aflatoxin intoxication), and by its mutational profile. Using a cohort enriched in HCCs with alcohol cirrhosis background, a recent extensive study found that mutations in Wnt/*β*-catenin partners predominate, 32.8% of HCCs being mutated in CTNNB1, 15.2% in AXIN1, and 1.6% in APC genes [32]. A pioneering work showed in 2001 that the HCCs mutated in CTNNB1 belong to a group of HCCs characterized by a low genomic instability and a better prognosis for patient survival [33]. This profile differs from that associated with p53 and AXIN1 mutations, that mainly consists in HCCs with a high chromosomal instability and a poor prognosis [34, 35]. Unexpectedly, AXIN1 mutations in HCCs do not activate efficiently *β*-catenin pathway, suggesting that its tumor suppressor function is mediated through other partnerships [36]. Interestingly, CTNNB1-mutated HCCs are less proliferative than nonmutated ones, suggesting that the genetic program by which *β*-catenin signalling triggers hepatocarcinogenesis is somehow different from that which is implemented following P53 mutations [37]. From studies performed in human HCCs and also from murine transgenic models in which Apc loss leads to *β*-catenin-activated liver tumors, several Wnt target genes have been described with a potential role in hepatocyte proliferation in a cancerous context: these are Cyclin D1, but not c-myc, the regenerating islet-derived 3- and 1-*α* genes (REG1A and REG3A), Tgf-*α* [15, 16, 38, 39]. However, the critical transcriptional targets by which *β*-catenin induces proliferation in liver cancers remain elusive.

### 3.2. Wnt in Quiescent Pericentral Hepatocytes

After the initial discovery of the oncogenic role for liver *β*-catenin, an unexpected Wnt signalling was detected in 2006 in a subset of quiescent hepatocytes located within the pericentral area [40]. It should be noted that a role of Wnt signalling in post-mitotic cells has been described not only in hepatocytes, but also in neurons and cardiomyocytes [5]. 

In mammals, the different metabolic functions of the liver, such as gluconeogenesis, glycolysis, glutamine synthesis, or urea formation are assumed by hepatocytes that differ in their location along the portocentral axis of the liver lobule, either near the portal triad (periportal, PP) or close to the central vein (pericentral, PC). This is the concept of metabolic zonation [41–43]. We found in 2006 that *β*-catenin is physiologically activated in pericentral hepatocytes [40]. This process is blocked by Dkk1 and is therefore Wnt-dependent even if the Wnt source around the central vein is not clearly identified, but could be of endothelial or stellate cell origin [44–46]. This *β*-catenin signalling is also due to the low amount in this area of APC, further defined as the “zonation-keeper” of the liver: as a consequence, its liver-specific loss enables a loss of zonation together with hepatocyte hyperproliferation, with a dramatic metabolic phenotype leading to the death of the mice [40]. This zonal patterning is due to *β*-catenin inducing the transcription of genes encoding metabolic enzymes in the pericentral area, whereas in the same zone it directly represses the transcription of genes encoding periportal enzymes or transporters ([42], for review). The quiescent liver is therefore an attractive model for Wnt research, allowing to decipher the molecular mechanism by which Wnt in G0 hepatocytes controls liver metabolism rather than proliferation.

### 3.3. Wnt in Liver Regeneration and Hepatocyte Proliferation

**Figure 2:**
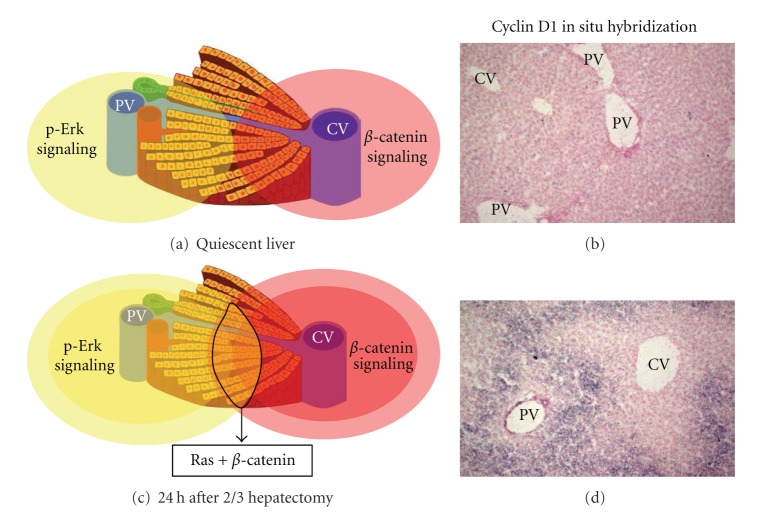
Hypothesis for a crosstalk between *β*-catenin and Ras signalling pathways to control hepatocyte proliferation. (a) In the quiescent liver, phospho-erk signaling is described as being periportal, while *β*-catenin is pericentral; (b) no cyclin D1 mRNA is detected in these conditions; (c) 24 h after 2/3 hepatectomy, there is an extension in the *β*-catenin and the ras activation territories. We now hypothesize that it could generate a common territory in which both which phospho-erk and *β*-catenin signalings are activated; (d) this can be exemplified by cyclin D1, which is a target of *β*-catenin, potentiated by ras signaling. Its localization after hepatectomy is restricted to the midlobular region. PV = portal vein; CV = central vein.

Consistent with the oncogenic role of *β*-catenin in the liver, the massive activation of *β*-catenin in more than 70% of hepatocytes in mice leads to hepatomegaly, partly due to hepatocyte proliferation [15, 16, 40, 47]. Conversely, liver weight has been shown to be 20% lower in adult mice with a liver-specific *β*-catenin inactivation than in wild-type mice [48]. Moreover, such *β*-catenin inactivation/overactivation murine models were submitted to two-third hepatectomies, and a role for *β*-catenin in liver regeneration has been firstly reported in 2006 [39, 48–51]. After hepatectomy, *β*-catenin activation extends from the pericentral area up to the midlobular hepatocytes. It takes place by 24 h after hepatectomy, and this corresponds to progression in G1: it induces at least Cyclin D1 and Tgf-*α* expressions [39]. But the distribution of proliferating hepatocytes is panlobular, while that of Cyclin D1 begins in the midlobular area, suggesting that *β*-catenin-dependent hepatocyte proliferation is dictated both by cell-autonomous and non-cell-autonomous mechanisms. Interestingly, the more distal pericentral hepatocytes do not initially express cyclin D1 in response to *β*-catenin, highlighting the resistance of PC hepatocytes to proliferate [52]. Moreover, we have shown that Ras signalling increased the *β*-catenin-dependent transcription of cyclin D1 further in hepatoma cells, confirming previous studies in colon cancer cell lines [4, 39]. As Ras/Erk1-2 is the predominant signalling pathway in the periportal area [53], we now hypothesize that it could cooperate with *β*-catenin in the midlobular hepatocytes to elicit an enhanced cyclin D1 transcription during liver regeneration (Figure 2). This hypothesis is supported by the fact that Ha-Ras and *β*-catenin signalling cooperate to accelerate liver tumorigenesis [54].

### 3.4. Wnt and Liver Stem Cells

Wnt signalling has a prominent role in stem cell biology, including self-renewal, pluripotency, and differentiation of both embryonic stem (ES) and somatic stem cells (reviewed in [55]). It was therefore attractive to search for an equivalent role in the liver, and the first publications in that field appeared by 2007.

It must be understood that liver homeostasis is not dictated by its self-renewal, due to the quiescence of the hepatocytes with a lifespan of 300–400 days [56]. Moreover, it is known that the mature quiescent hepatocyte is able to re-enter into the cell cycle and to self-renew without the need for a stem cell. However, some particular cells located in the vicinity of the portal triad within the so-called Herring canal are a reservoir for regeneration in particular contexts of liver diseases or for specific oncogenesis [57]. Several signalling pathways have been shown to play an important role in the emergence, expansion, and differentiation of these transiently amplifying progenitor cells, also referred to as oval cells [58]. An active Wnt signalling has been found during progenitor cell-mediated regeneration of the liver (drug-induced models in which hepatocyte proliferation is blocked, forcing the putative stem cells to engage into the cell cycle) [56, 59, 60]. 

## 4. Perspectives: How Hepatic Wnt Signalling Impacts the Cell Cycle?

The role of Wnt in the adult liver is paradoxical, due to its dual role as patterning its metabolic zonation, while being engaged in liver-cell proliferation both physiologically in regeneration processes, and pathologically during oncogenesis. 

In fact, the balance between quiescence and proliferation has to be fine-tuned in order to avoid either a fatal loss or tissue regeneration or neoplasia [61, 62]. So it is attractive that Wnt signalling could be such a sensor, perfectly adapted to liver needs. The dissection of the various modes whereby Wnt signalling impacts G1 in the liver and the identification of the molecular network that shifts Wnt from a metabolic to a mitogenic output may help designing specific cancer therapies.

## Abbreviations

CK1: Caseine kinase 1
GSK3: Glycogen synthase kinase 3
APC: Adenomatous polyposis coli
LRP5/6: Lipoprotein-related protein 5/6
Lef/Tcf: Lymphoid enhancer factor/T-cell factor
Rb: Retinoblastoma
c-Myc: Myelocytomatosis oncogene
IGF/AKT: Insulin growth factor/Akt
TOR: Target of rapamycin
TSC2: Tuberous sclerosis 2
MT: Microtubule
LGR5: Leucine rich repeat containing G protein-coupled Receptor 5
CDK14: Cyclin-dependent kinase 14
HCC: Hepatocellular carcinoma
REG1A/REG3A: Regenerating islet-derived 1 alpha/3 alpha
PC: Pericentral
PP: Periportal
TGF*α*: Transforming growth factor alpha
Ras: Rat sarcoma viral homolog
ERK: Extracellular regulated MAP kinase
PV: Portal vein
CV: Central vein.
